# Emerging from the Darkness. Sudden Cardiac Death in Cardiac Amyloidosis

**DOI:** 10.31083/j.rcm2310345

**Published:** 2022-10-14

**Authors:** Valeria Cammalleri, Valeria Maria De Luca, Giorgio Antonelli, Ombretta Annibali, Annunziata Nusca, Simona Mega, Myriam Carpenito, Danilo Ricciardi, Fiorella Gurrieri, Giuseppe Avvisati, Gian Paolo Ussia, Francesco Grigioni

**Affiliations:** ^1^Fondazione Policlinico Universitario Campus-Biomedico, Operative Research Unit of Cardiovascular Science, 00128 Roma, Italy; ^2^Department of Cardiovascular Science, Università Campus Bio-Medico di Roma, 00128 Roma, Italy; ^3^Department of Haematology, Università Campus Bio-Medico di Roma, 00128 Rome, Italy; ^4^Laboratory of Medical Genetics, Università Campus Bio-Medico di Roma, 00128 Rome, Italy

**Keywords:** implantable cardioverter defibrillator, sudden cardiac death, cardiac amyloidosis, monoclonal immunoglobulin light chains, transthyretin amyloidosis, ventricular arrhythmia

## Abstract

Cardiac amyloidosis (CA) manifests as infiltrative cardiomyopathy with a hypertrophic pattern, usually presenting with heart failure with a preserved ejection fraction. In addition, degenerative valvular heart disease, particularly severe aortic stenosis, is commonly seen in patients with CA. However, amyloid fibril deposition might also infiltrate the conduction system and promote the development of electrical disorders, including ventricular tachyarrhythmias, atrio-ventricular block or acute electromechanical dissociation. These manifestations can increase the risk of sudden cardiac death. This review summarises the pathophysiological mechanisms and risk factors for sudden cardiac death in CA and focuses on the major current concerns regarding medical and device management in this challenging scenario.

## 1. Introduction

Amyloidosis is a disorder of protein conformation that results in the deposition of insoluble fibrils in tissues. Increased deposition of amyloid can lead to organ failure and death. Various proteins can aggregate as amyloid *in vivo* but few can infiltrate the myocardium, causing severe cardiac dysfunction [1, 2]. The current diagnosis of cardiac amyloidosis (CA) usually refers to the deposition of fibrils composed of monoclonal immunoglobulin light chains (AL) or misfolded monomers of transthyretin (ATTR), secondary to hereditary (ATTRh) or acquired wild-type (ATTRwt) mutations. Initially considered a rare disease, recent data have revealed an increasing trend in the diagnosis, suggesting that CA is underestimated [2, 3].

In systemic AL amyloidosis, a plasma cell clone or, less commonly, a lymphoplasmacytic or marginal zone lymphoma produces a toxic light chain (LC) that causes tissue damage and organ dysfunction by forming amyloid fibrils. In contrast, localized deposition of LCs causes nodules to develop in the skin, as well as the respiratory, urinary and gastrointestinal tracts, with local symptoms and a benign course that is usually managed with local treatment [4]. In systemic AL amyloidosis, the plasma cell clone is usually small (median infiltrate, 10%) and presents (11;14) and gain 1 (q21) in approximately 50% and 20% of clones, respectively, whereas high-risk aberrations are uncommon [5, 6].

In ATTRh, aminoacid substitution in the transthyretin (*TTR*) gene sequence (composed of 4 exons on chromosome 18) results in a destabilisation and misfolding process that leads to amyloidogenesis. In contrast, ATTRwt is a sporadic disease characterised by a normal *TTR* gene sequence, although the causes of TTR misfolding that occurs with age are unclear. In the hereditary form, multiple organs are affected by the deposition of amyloid fibrils, resulting in a variable phenotypic appearance, such as sensorimotor polyneuropathy, gastrointestinal tract disorders and cardiac and renal failure. Some ATTR variants are associated with specific symptoms ranging from pure polyneuropathy to mixed neurologic and cardiac presentation to selective cardiac involvement, as well as different ages of onset [7, 8]. In contrast, ATTRwt is characterized by more cardiac and soft tissue involvement alone [9].

At the cardiac level, amylogenic proteins accumulate in the extracellular space, distorting the cardiac myocytes and valvular systems. This results in infiltrative cardiomyopathy with a hypertrophic pattern, usually presenting with heart failure with preserved ejection fraction and degenerative aortic stenosis, particularly with a low-flow-low-gradient pattern [10, 11, 12]. Furthermore, the infiltrative involvement of other endocardial tissues can lead to multiple valvular heart diseases [13, 14]. In addition, the deposits of amyloid fibrils can infiltrate the conduction system, enhancing the genesis of rhythm disturbances [10, 15, 16].

Despite the phenotypic differences with more amyloid infiltration in ATTR than in AL, the haematological form of amyloidosis has worse prognosis with a high rate of sudden cardiac death (SCD). In particular, the prevalence of SCD has been estimated to be approximately 33% in the first 3 months after AL diagnosis [7, 17]. Nevertheless, the current scientific evidence does not support specific recommendations for preventing SCD in patients suffering from CA.

This review provides an overview of the pathophysiological mechanisms and risk factors of SCD in CA and focuses on the major current concerns about medical and device management of amyloidotic patients.

## 2. Sudden Cardiac Death in CA

Heart involvement is the major determinant of survival in patients with amyloidosis, and ‘sudden death’ occurs in approximately two-third of patients with CA [18, 19]. Extensive myocardial infiltration with the involvement of the conduction system suggests that various processes may be responsible for SCD, such as ventricular tachyarrhythmias, atrio-ventricular block or electromechanical dissociation [20, 21, 22, 23, 24]. These matters are supported by pathologic findings of amyloid deposits within the conduction system and fibrosis of the sinoatrial node and bundle branches [25, 26].

Other proposed mechanisms driving electrophysiological manifestations of CA involve intramural coronaries, microvascular ischemia or patchy myocardium infiltration of amyloid fibrils, causing the development of anatomical re-entrant circuits responsible for ventricular arrhythmia [27, 28]. Moreover, AL amyloid fibrils are highly cytotoxic to the ventricular myocardium, explaining why ventricular arrhythmias appear more frequently in AL than in ATTR. Preclinical models and clinical observations of rapid cardiac improvement after a decline in LC concentration disclosed a direct cardiotoxic effect of the circulating precursor in the AL population [29, 30]. Survival also depends on hematologic response because LCs seem to be the agents directly causing organ dysfunction. If the disease is not treated promptly and effectively, organ dysfunction progresses and eventually leads to death. In addition, the accumulation of amyloid fibrils induces inflammation and therefore generates oxidative stress in the cardiomyocytes, which causes fibrosis, even though myocardial scarring and fibrosis are less common in CA than in ischemic or non-ischemic cardiomyopathy [17, 31, 32, 33]. Consequently, inflammatory cell injury, cell damage and disconnection of myocytes by amyloid fibrils justify electrophysiological disorders.

In addition, therapeutic treatment can influence arrhythmic phenomena. Dexamethasone, used for patients with AL, potentiates fluid retention and promotes arrhythmias, resulting in an arrhythmogenic ventricular substrate [34]. Similarly, cardiotoxicity induced by a high dose of chemotherapy used in patients with AL (such as cyclophosphamide and bortezomib) can result in ventricular tachycardia (VT), secondary to myocardial dysfunction [35, 36]. The simultaneous presence of valvular heart disease (such as severe aortic valve stenosis and significant mitral or tricuspid disease) might be responsible for a lower threshold in developing arrhythmias.

Although published studies have suggested that death is often attributed to electromechanical dissociation, small series have reported successful defibrillation in individual patients with implantable cardioverter defibrillators (ICDs) [19, 37, 38]. Non-sustained ventricular tachycardia (NSVT) has been observed with a prevalence of 5–27% in the routine monitoring of patients with AL, reaching 100% during the stem-cell transplant period [7, 39, 40, 41]. Conversely, fewer publications have reported the rate of ventricular arrhythmias in the ATTR population, but the prevalence has been estimated to be approximately 17% [42]. Nevertheless, the presence of ventricular arrhythmias does not seem to predict SCD in patients with CA [7, 28, 43]. Table 1 (Ref. [7, 19, 37, 39, 40, 41, 42, 43, 44]) summarises the studies that have investigated ventricular arrhythmias in patients with amyloidosis.

**Table 1.** **Published data on ventricular arrhythmias in cardiac amyloidosis**.

Study author	Number of patients	Type of amyloidosis	% of ventricular arrhythmias	Method of study
Dubrey *et al*. [44]	232	AL	26.7%	24-h Holter monitoring
Palladini *et al*. [39]	51	AL	18%	24-h Holter monitoring
Hörnsten *et al*. [42]	30	ATTR	16.7%	24-h Holter monitoring
Murtagh *et al*. [40]	127	AL	5%	Electrocardiogram
Kristen *et al*. [19]	19	AL	11%	ICD
Goldsmith *et al*. [41]	24	AL	100%	Telemetry
Varr *et al*. [37]	31	AL and ATTR	74%	ICD, pacemaker, telemetry
Sayed *et al*. [7]	20	AL	5%	Implantable loop recorder
Hamon *et al*. [43]	45	AL and ATTR	27%	ICD

AL, light chain amyloidosis; ATTR, transthyretin amyloidosis; ICD, implantable cardioverter defibrillator.

## 3. Risk Factors for SCD

Recent studies aiming to identify predictors of SCD in patients with CA have reported ambiguous results. In patients with AL-CA, Kristen *et al*. [19] identified the following risk factors for electromechanical dissociation: multiple ventricular ectopic beats, low-voltage electrocardiogram (ECG) patterns, increased left ventricular wall thickness and high levels of N-terminal prohormone of brain natriuretic peptide (NT-pro-BNP). Furthermore, risk factors for VT genesis have been found in patients with increased left ventricular end-systolic dimension, left ventricular ejection fraction (LVEF) <30%, QRS duration >125 ms, age >65 years, history of heart failure (HF) and need for diuretic drugs [45, 46]. In addition, syncope is considered an independent risk factor for SCD [47], although it is an unspecific symptom in patients with CA, being secondary to conduction disturbances, vagal events, orthostatic hypotension, dysautonomia or intake of diuretic drugs or vessel dilators. Moreover, in the AL population, the occurrence of NSVT is associated with worse survival before stem-cell transplant [48].

The data obtained from cardiac magnetic resonance (CMR) imaging studies have shown that the increase in late gadolinium enhancement predicts death (HR 5.4; 95% CI, 2.1–13.7; *p *< 0.0001) as an expression of a heart failure pattern and surrogate of possible lethal arrhythmias. Moreover, T2 imaging of myocardial edema is inextricably linked to arrhythmogenic potential, as an expression of active inflammation [49, 50].

In contrast, data from electrophysiological studies (EPSs) have identified abnormalities of ventricular conduction and repolarisation as markers of increased risk of developing VT and SCD [16, 51]. In particular, the lengthening of the His-Ventricular (HV) interval >55 ms and the presence of late potentials are independent predictors of SCD in AL-CA [16]. Nevertheless, VT inducibility on EPS does not predict SCD in this subset of patients [16].

However, an EPS for assessing distal conduction disease caused by CA and VT inducibility can be used to stratify high-risk populations to manage catheter ablation strategies or ICD implantation [52].

Interestingly, different conduction abnormalities have been observed in the different subtypes of CA, especially in the AL population, when compared to ATTR, in the form of lower epicardial signal amplitude (1.07/0.46 versus 1.83/1.26 mV, *p* = 0.026), greater epicardial signal fractionation (4.00/1.75 versus 3.00/1.00, *p* = 0.019) and slightly higher dispersion of repolarisation (187.6/65 versus 158.3/40 ms, *p* = 0.062), without significant differences in the standard 12-lead ECG [51]. These data can be interpreted as a higher risk of developing ventricular arrhythmias in the AL population.

## 4. Medical Therapy

**Fig. 1.**
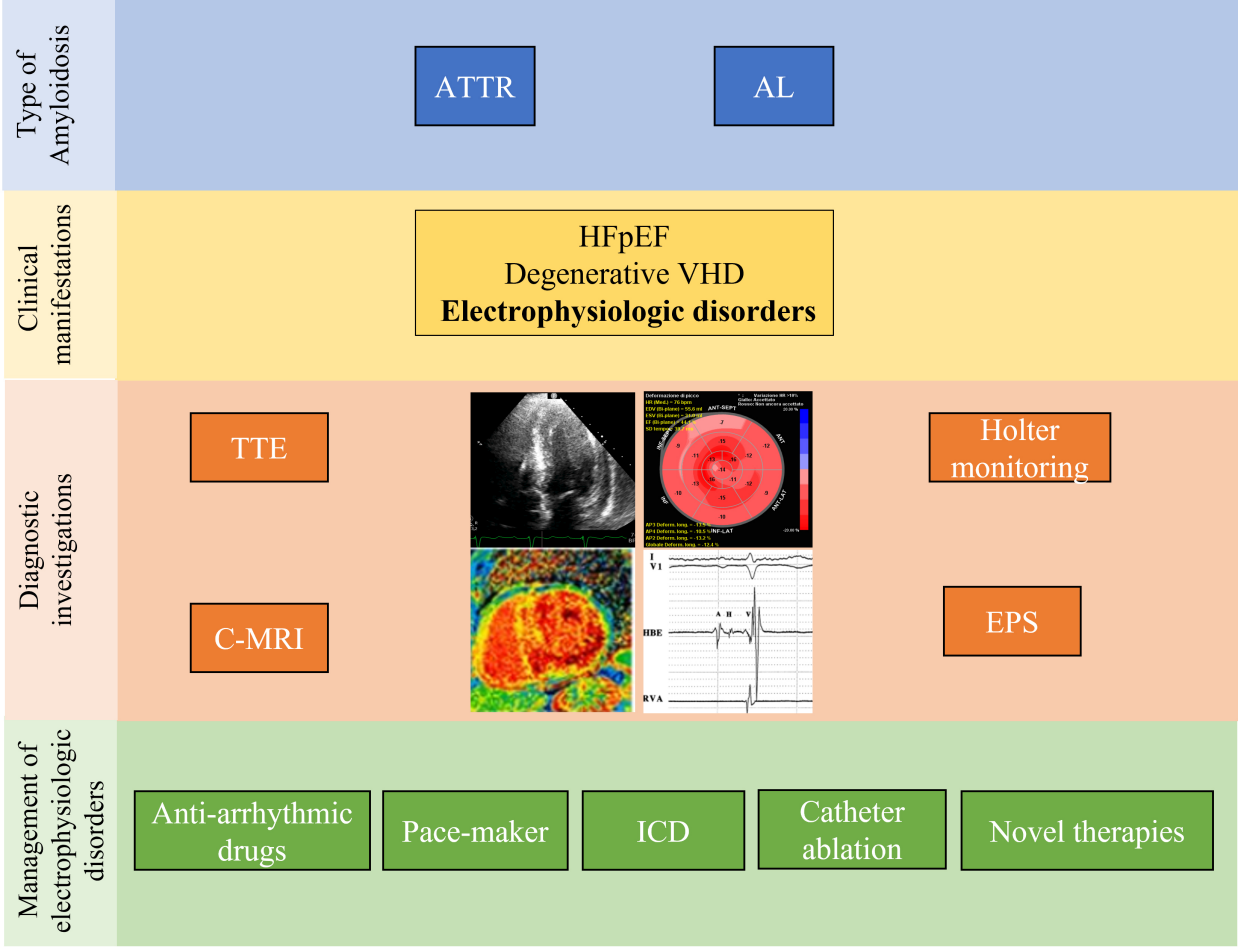
**Management of electrophysiologic manifestations of CA**. ATTR, transthyretin amyloidosis; AL, light chain amyloidosis; HFpEF, heart failure with preserved ejection; VHD, valvular heart disease; TTE, transthoracic echocardiogram; C-MRI, cardiac magnetic resonance imaging; EPS, electrophysiological study; ICD, implantable cardioverter defibrillator.

The management of CA patients at high risk of SCD entails different strategies derived from standard recommendations for HF and for conventional cardiomyopathies [53, 54, 55, 56] (Fig. 1).

In effect, patients with CA tolerate standard medical therapies poorly, as these therapies can lead to a low-output state and worsening haemodynamics. The restrictive physiology at CA is responsible for a relatively fixed stroke volume; consequently, the maintenance of the cardiac output depends strictly on the heart rate and diastolic filling of the left ventricle. Therefore, betablockers and calcium channel blockers, reducing high heart rates, diastolic filling time and, consequently, cardiac output are poorly tolerated [57, 58, 59]. Moreover, digoxin can be cardiotoxic in patients with CA and compromise myocardial contractility [33, 59]. Amiodarone can promote prolongation of the correct QT interval (QTc) and torsades de pointes; worsening of systolic function due to the inherent beta-antagonist activity and complete heart blockage, more in patients with CA than in those without CA (43.8% versus 30.0%, *p *< 0.0001) [60].

## 5. Device Therapy

Considering the high risk of conduction system disease, permanent pacemakers are often implanted in patients with CA, most commonly in ATTRwt-CA, followed by ATTRh-CA and AL-CA [24, 61, 62]. Moreover, the disease often progresses, resulting in an increased burden of right ventricular pacing, which can have harmful consequences. In a study of patients with ATTR-CA and cardiac implantable electronic devices, a pacing burden >40% was associated with adverse clinical and echocardiographic outcomes, including worsening New York Heart Association (NYHA) functional class, LVEF and mitral regurgitation [62]. Considering that patients with CA poorly tolerate pacing-mediated intraventricular and interventricular dyssynchrony due to their restrictive physiology, biventricular pacing should be chosen when pacing is indicated [24]. To date, prophylactic pacemaker implantation for preventing major cardiac events in patients with CA remains uncertain [61].

Similarly, although ICDs are often implanted for primary or secondary prevention according to current guidelines, their role in CA remains controversial, and a survival benefit has not been proven: many patients have a survival of <1 year, which is usually a contraindication to ICD implantation. SCD in these patients occurs mainly from electromechanical dissociation or asystole rather than ventricular arrhythmias. In addition, amyloid infiltration in the myocardium can lead to high defibrillation thresholds, making ICD therapy unsuccessful. ICD can detect and can cause early termination of a non-sustained ventricular arrhythmias that would not have led to sudden death, or it can be pro-arrhythmic because antitachycardia pacing accelerates slower ventricular rhythms into the ventricular fibrillation zone, leading to ICD shock [63, 64]. Finally, the termination of sustained and potentially fatal ventricular arrhythmias in patients with advanced HF due to CA can avoid arrhythmic death but cannot prevent death due to pump failure or electromechanical dissociation [63].

Several attempts have been made to create risk scores for identifying the best patients who can benefit from device therapy. Varr *et al*. [37] proposed the Stanford Amyloid Center’s ICD implantation criteria for appropriate implantation in patients with CA, who had a good quality of life, NYHA functional class <IV, history of non-postural syncope and NSVT and sustained VT. Nevertheless, several concerns exist regarding the absolute advantage that ICDs can confer to patients with CA and which type of amyloidosis can effectively benefit from ICD placement. Real-world data suggest that CA patients with NSVT are best treated with an ICD as secondary prevention [37, 65]. Conversely, a study by Hamon *et al*. [43] involving the majority of patients with ATTR demonstrated that ICD insertion was most appropriate in patients with less advanced cardiac remodeling, even though the burden of ventricular arrhythmia did not impact the overall survival. Likewise, Kim *et al*. [66] demonstrated that ICD therapy did not prolong survival in CA patients when compared to patients without ICD (44 versus 40 months, *p* = 0.76), and higher mortality was observed in patients with CA despite ICD therapy when compared to patients without CA (40.5 versus 26.2%, *p *< 0.045).

In the case of primary prevention, the decision to implant an ICD requires strict patient selection criteria. For example, in patients with low LVEF and progressive HF, ICD implantation would not provide further benefits because of the increased risk of electromechanical dissociation [19]. Conversely, in AL amyloidosis, ICD implantation can well be recommended in patients awaiting cardiac transplantation or left ventricular assist devices [67].

To date, published results are conflicting, and there is no consensus on absolute recommendations. Consequently, a multidisciplinary team approach and shared decision-making strategy between physicians and patients are critical when considering the implantation of an ICD in the CA population.

## 6. Future Perspective

Stratifying a subgroup of CA patients with a high arrhythmic risk, besides having future implications in the daily clinical approach, can yield promising results in reducing the same risk in patients treated with new pharmacological approaches. In particular, disease-modifying therapies targeting the production of amyloid precursor protein or the assembly of amyloid fibrils can treat the process of amyloid deposition.

In AL setting, amyloidosis treatment is usually risk-adapted, considering the severity of organ involvement, characteristics of the clone and comorbidities, with the aim to deliver the most rapid and effective therapy patients can safely tolerate. Delicate up-front therapy can sometimes trigger early improvement in organ dysfunction, allowing for a subsequent, more aggressive treatment. In approximately one-fifth of patients, autologous stem-cell transplantation is considered up-front or after bortezomib-based conditioning. Bortezomib can improve the response depth after transplantation and is the backbone of treatment for patients who are not eligible for transplantation. The combination of daratumumab and bortezomib is emerging as a novel standard of care in AL amyloidosis. Early and profound reductions of the amyloid LC are associated with the greatest chance of organ improvement and prolongation of progression-free and overall survival [68]. However, recent trials of immunotherapies targeting amyloid deposits have failed, in particular for the anti-fibril antibody NEOD001 and the combination of the amyloid P component (which targets small-molecule miridesap) and dezamizumab [68]. Only one anti-amyloid fibril antibody, CAEL-101, is still under evaluation and has recently shown encouraging results in the 1a/b phase study. Sixty-three percent of patients with cardiac, renal, hepatic, gastrointestinal or soft tissue involvement had a therapeutic response to mAb CAEL-101, as evidenced by serum biomarkers, including natriuretic peptides, and objective imaging modalities [69]. This suggests that CAEL-101 can modify the course of AL CA by removing amyloid fibrils in cardiac tissue.

Comparably, there are novel therapeutic options for ATTRwt and ATTRh, namely TTR stabilizer (tafamidis) and TTR production suppressor (inotersen and patisiran) [2, 70]. In a previously published randomized study, tafamidis resulted in clinical benefits and increased cardiovascular outcomes in patients with ATTR-CA, reducing all-cause mortality and cardiovascular hospitalizations at 30 months and improving echocardiographic parameters [71]. In addition, in a recently published CMR study, Rettl *et al*. [72] demonstrated that tafamidis delays the progression of myocardial infiltration in patients with ATTR-CA, as measured by T1 mapping of the extracellular volume, along with functional and clinical improvements. Similarly, data obtained from the APOLLO trial in the cardiac subpopulation of patients with ATTRh amyloidosis showed that, compared to placebo, patisiran reduced left ventricular wall thickness, increased end-diastolic volume and cardiac output and reduced adverse cardiac outcomes (rates of cardiac hospitalizations and all-cause death) at 18 months [73]. Based on these pathophysiological findings, we expect that this class of therapies can reduce the arrhythmia burden in the CA population. In addition, ICD implantation can be increasingly considered, as the survival of patients with CA improves with this class of disease-modifying therapies.

However, further analyses are required to define their efficacy in CA-associated arrhythmias. Additionally, in the context of hereditary forms, the observation of large populations for long periods can lead to identifying genetic mutations at higher risk or with a protective factor regarding ventricular arrhythmias, as demonstrated previously for hypertrophic cardiomyopathy, Brugada syndrome and long-QT syndrome.

Therefore, starting from a pathophysiological assumption, using clinical knowledge combined with the most modern imaging techniques, it is essential to engage correct resources in this special population deemed at high risk for SCD.

## 7. Conclusions

Amyloid infiltration into the conduction system enhances the genesis of rhythm disturbances, including fatal ventricular arrhythmias and SCD. Current pharmacological anti-arrhythmic therapies are poorly tolerated by patients with CA, and there are no robust recommendations on the management of ventricular arrhythmias in this subset of patients. Furthermore, the benefits of ICD implantation are highly variable according to the different clinical stages of the disease. Therefore, further studies are needed to create a standardized diagnostic algorithm and an appropriate treatment strategy for this special population.
